# Pilot-scale evaluation of a dynamic body-feed filtration system for primary clarification of snake antivenoms produced by the caprylic acid method

**DOI:** 10.1016/j.toxcx.2024.100202

**Published:** 2024-08-13

**Authors:** Andrés Sánchez, Maykel Cerdas, Jairo Gutiérrez, Mariángela Vargas, Álvaro Segura, María Herrera, Stephanie Chaves-Araya, Ronald Sánchez, Mauren Villalta, Gina Durán, Adriana Sánchez, Gabriela Solano, Daniel Cordero, Paola Sánchez, José María Gutiérrez, Guillermo León

**Affiliations:** Instituto Clodomiro Picado, Facultad de Microbiología, Universidad de Costa Rica, San José, Costa Rica

**Keywords:** Caprylic acid, Diatomite, Dynamic body-feed filtration, Equine plasma, Snake antivenom

## Abstract

The performance of dynamic body-feed filtration (DBF) in the removal of bulky solids produced during the manufacturing of snake antivenoms using the caprylic acid method was evaluated. For this purpose, diatomites with different filterability properties were compared in a bench-scale study to assess their effectiveness in removing the precipitated material formed after the addition of caprylic acid to equine hyperimmune plasma. C1000 diatomite at a concentration of 90 g/L of precipitated plasma showed the best performance. Then, the process was scaled up to three batches of 50 L of hyperimmune horse plasma. At this pilot scale, 108 ± 4% of the immunoglobulins present following plasma precipitation were recovered after DBF. The antivenoms generated using this procedure met quality specifications. When compared to open filtration systems commonly used at an industrial scale by many antivenom manufacturers, DBF has a similar yield and produces filtrates with comparable physicochemical characteristics. However, DBF ensures the microbiological quality of the primary clarification in a way that open systems cannot. This is because: 1) DBF is performed in a single-use closed device of depth filters which prevents microbial contamination, and 2) DBF removes bulky material in few minutes instead of the more than 24 h needed by open filtration systems, thus reducing the risk of contamination. It was concluded that DBF is a cost-effective, easily validated, and GMP-compliant alternative for primary clarification following caprylic acid precipitation of plasma in snake antivenom production.

## Highlights

• Caprylic acid precipitation is a recognized method for producing antivenoms.
• The removal of bulky solids is a challenging step at an industrial scale.
• A pilot-scale evaluation of dynamic body-feed filtration was conducted.
• Dynamic body-feed filtration is suitable for antivenom production.

## Graphical abstract

**Figure:**
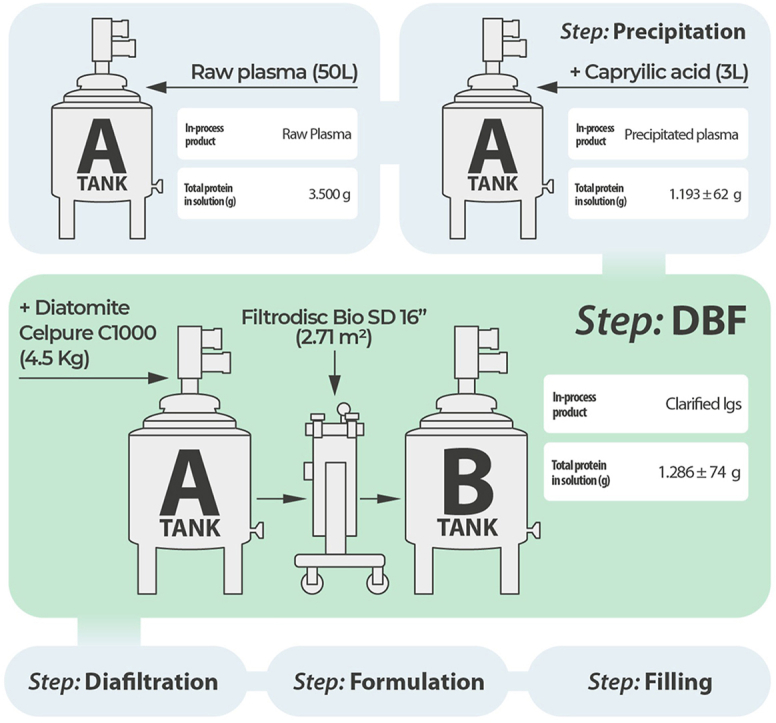

## 1 Introduction

The caprylic acid precipitation method is one of the procedures recommended by the World Health Organization (WHO) for the primary purification of the immunoglobulins (or immunoglobulin fragments) used as active substance in the formulation of snake antivenoms (WHO, 2017). In general, this method is based on the ability of caprylic acid to denature and precipitate non-immunoglobulin proteins present in the plasma of animals immunized with snake venoms (Steinbuch and Audran, 1969; dos Santos et al., 1989). After removing the precipitated material, the purified immunoglobulins remaining in solution undergo further polishing, sterilization, dispensing into borosilicate vials, and stabilization according to the formulation design (León et al., 2018).

Current literature contains numerous reports of the use of the caprylic acid method to produce antivenoms for different geographical regions (dos Santos et al., 1989; Rojas et al., 1994; Gutiérrez et al., 2005; Fernández et al., 2010; Vargas et al., 2011; Simsiriwong et al., 2012; Al-Abdulla et al., 2013; Guidolin et al., 2016; Villalta et al., 2016; Kishmiryan et al., 2021). However, most of these reports correspond to bench-scale procedures, which do not fully reflect the conditions present throughout industrial-scale manufacturing. Normally, scaling up processes in accordance with the current Good Manufacturing Practices (cGMPs) for the Pharmaceutical Industry presents a series of tasks that must be addressed (Xia et al., 2016).

One of the most challenging stages to scale up the caprylic acid method is the removal of the bulky solids generated during the protein plasma precipitation. As is customary in bioprocesses, primary clarification plays a critical role in the process because it significantly impacts the product recovery and the subsequent downstream purification (Cherradi et al., 2018). Therefore, it must be optimized and validated to meet the product specifications and manufacturing constraints (WHO, 2017).

The selection of the clarification technique depends on the quantity and nature of solids to be removed, and the properties of the fluid of interest. Some antivenom producers use continuous flow centrifugation for the separation of solids generated during the purification steps (Raweerith and Ratanabanangkoon, 2003). Despite its efficient operational performance, centrifugation has the disadvantage of requiring a large economic investment, high maintenance costs, and high energy consumption (Cherradi et al., 2018), which could render this strategy unattainable for antivenom manufacturers in middle- and low-income countries.

Alternatively, there are filtration methods available for the clarification of immunoglobulins, such as microfiltration operated in normal flow filtration (NFF) or tangential flow filtration (TFF), or single-use depth filters operated as NFF (Van Reis and Zydney, 2001). These methods are easier to scale up and implement on the industrial scale. Nevertheless, their main drawbacks are associated with filter blocking and turbidity breakthrough, leading to a decrease in flow rates and early increase in the system pressure (Sharma et al., 2017).

Another option is the dynamic body-feed filtration (DBF). In this separation technology, the in-process product (i.e., precipitated plasma) is blended with a filter aid (i.e., diatomite) to form a paste. During the filtration, this paste gradually deposits on the surface of the filter, forming a cake that constitutes the actual filter medium. The porosity, high surface area, and relative hardness of the filter aid prevent the collapse of compressible solids and promote the formation of flow channels inside the cake, increasing the filterability through the precipitate (Nejatishahidein and Zydney, 2021). Current commercial DBF options consist of closed single-use systems, which reduce operational costs, cross-contamination risks, and validation efforts.

DBF could be an alternative for laboratories that make significant contributions to providing vulnerable communities with antivenoms but need to improve the quality of their processes and often lack the necessary resources to acquire expensive equipment. Many of these laboratories conduct primary clarification using open systems of filter paper placed on funnels and glass bottles, as in bench-scale reports. While in bench-scale open filtration takes a few minutes (Rojas et al., 1994), it takes up to 24 h at an industrial scale. During this time, the in-process products are exposed to the risk of microbial contamination and growth. Implementation of DBF could contribute to the improvement in the quality of available antivenoms, some of which are known to have issues related to contamination with microbial products (Solano et al., 2024).

In this study, the effectiveness of diatomites with different filterability properties in assisting the removal of solids generated during the precipitation of equine plasma by adding caprylic acid was compared in bench-scale experiments. Different concentrations of the diatomite with the best profile were then compared to enhance the process yield. The optimized conditions were scaled up to three pilot batches of plasma (50 L each). The performance of the pilot-scale process and the quality of the obtained antivenoms were assessed to determine the suitability of DBF for the GMP production of snake antivenoms.

## 2 Materials and methods

All procedures performed in this study using animals meet the International Guiding Principles for Biomedical Research Involving Animals (CIOMS, 2012) and were approved by the Institutional Committee for the Care and Use of Laboratory Animals of Universidad de Costa Rica (approval code CICUA 202–2020).

### 2.1 Snake venoms and equine plasma

Venoms of adult specimens of *Bitis arietans* (batch #322.061), *Echis ocellatus* (batch #216.031) and *Naja nigricollis* (batch #616.031) were purchased from Latoxan (Portes-dès Valence, France). Freeze-dried venoms were acquired from the supplier and stored at −40 °C until use. Solutions of venoms were prepared right before use. The starting raw plasma was collected from horses immunized towards the venoms of *B. arietans*, *E. ocellatus*, and *N. nigricollis* (Gutiérrez et al., 2005). Plasma collection was conducted 12 days after the last venom booster. The horses underwent three consecutive days of bleeding followed by self-transfusions of resuspended erythrocytes on the second and third days, according to the method outlined by Huertas and colleagues (2023). The bleeding process utilized a system of PVC blood bags and citrate dextrose solution (ACD; citric acid 0.093 mol/L, sodium citrate 0.197 mol/L, dextrose 0.6 mol/L) as anticoagulant.

### 2.2 Bench-scale optimization of grade and amount of diatomite

After adding 6 mL of caprylic acid (Sigma, C-2875) at room temperature, 100 mL of plasma was precipitated. The percentage of solids in the precipitated plasma was determined by centrifuging a 10 mL sample at 1000×*g* for 15 min. The percentage of solids was determined by volume. For example, if the volume of solids was 3 mL, then the percentage of solids in the sample was 30%. After 1 h of vigorous stirring, the corresponding amount of USP-NF grade diatomite with varying filterability properties (e.g., Celpure C65, C100, C300, or C1000; Filtrox AG, St.Gallen, Switzerland) was added. The quantity of diatomite per filtration was calculated using the following equation:

$$Diatomite\;mass\;\left(Kg\right)=\frac{\%\;solids}{100}\;x\;precipitated\;plasma\;volume\;\left(L\right)\;x\;0.3$$

The mixture was stirred for 30 min. The paste formed by precipitated plasma and diatomite was then removed using DBF (Filtrodisc BIO SD 2”, CH 09P; 0.002 m^2^; Filtrox AG, St.Gallen, Switzerland). The filtrate trapped in the system was flushed with 50 mL of a 0.3 g/dL NaCl solution. The filtration flux was calculated by measuring the weight of the filtrate collected at various time points during filtration. The filtration pressure was measured using a manometer, and the turbidity and total protein of the filtrate were determined as described in section 2.4. The yield percentage of DBF was calculated using the following equation:

$$Yield\;percentage\;\left(\%\right)=\frac{total\;protein\;after\;DBF}{total\;protein\;after\;CA\;precipitation}\;x\;100$$

The performance of filtration and filtrate characteristics were compared to identify the diatomite with the best filterability profile. The bench-scale filtration was optimized by comparing various amounts of the selected diatomite under the experimental conditions described above. All experiments were conducted in duplicate.

### 2.3 Pilot-scale assessment of DBF

Three independent pilot batches of plasma were processed. In each batch, 3 L of caprylic acid (Sigma, C-2875) were slowly added to 50 L of undiluted plasma with vigorous stirring to achieve a final concentration of 5.7% (v/v). After 60 min of mixing at room temperature (18–20 °C), 4.5 kg of diatomite (Celpure®, C1000) was added. The mixture was stirred for an additional 30 min. Then, with the help of a peristaltic pump (Flowmaster FMT300, Ismatec, Zurich, Switzerland) fitted with a 15.9 mm internal diameter tube (PharmaMed® BPT, Saint-Gobain, France), the mixture was passed through a DBF system (Filtrodisc Bio SD 16” double module, CH09P; Filtrox AG, St.Gallen, Switzerland) equipped with 2.71 m^2^ of cellulose PURAFIX® PF P filter sheets, at a rate of 2.0 L/min. The filtrate trapped in the system was flushed with 100 L of a 0.3 g/dL NaCl solution. The filtrate underwent dialysis using 18.6 m^2^ of a 30 kDa cutoff ultrafiltration membrane (CDUF050LT, Helicon® SS50 Spiral Cartridge, Millipore, Darmstadt, Germany) and was formulated at pH 7.0–7.2, with 0.30–0.35 g/dL NaCl (Sigma, S-1679), 0.15–0.20 g/dL phenol (Sigma, P-9346), and 4.0–5.0 g/dL sucrose (Sigma, S-8501). After sterilization through membrane filtration (Sartorius, 5102507H2), the antivenoms were aseptically filled into sterile 10 mL borosilicate vials and stabilized by lyophilization (freezing at −40 °C for 2 h, annealing at −10 °C for 5 h, then freezing again at −40 °C for 2 h, sublimation at a shelf temperature of −10 °C and a chamber pressure of 200 mTorr for 70 h, and desorbed at 30 °C for 5 h; Herrera et al., 2014). Samples of in-process and final products were collected to assess the performance of the DBF and the quality of the final antivenoms. Ten milliliters of a 0.6 g/dL NaCl solution were used to reconstitute each vial of lyophilized antivenom for the subsequent tests.

### 2.4 Physicochemical quality control

Residual moisture was determined using Karl Fisher titration (Herrera et al., 2014). Reconstitution time was visually determined as the time needed for the lyophilized antivenom to completely dissolve when gently agitated by hand (Herrera et al., 2014). Turbidity was determined by turbidometry (Model, 2020 Turbidimeter, La Motte, USA) and reported in nephelometric turbidity units (NTU). The content of immunoglobulin monomers was determined using high-performance liquid chromatography (HPLC) on an Agilent 1100 series system (Agilent Technologies) with an Agilent Bio SEC-3 300a column (7.8 × 300 mm). A 150 mM phosphate buffer (pH 7.0) was used as the eluent, with a flow rate of 1.0 mL/min and detection at 214 nm. The pH was measured using potentiometry (USP-NF, 2024a). NaCl content was determined using a volumetric method (USP-NF, 2024b). Phenol concentration was measured using the 4-aminoantipyrine spectrophotometric method (Lacoste et al., 1959). Sucrose levels were measured using HPLC with refractometry detection (Agilent 1220 Infinity II HPLC with 1260 Infinity II RID module, Zorbax NH2 column). The osmotic concentration of solutes in the antivenoms was determined using osmometry (Advanced Instruments, Model 3320). Total protein was determined by the Biuret method (Gornall et al., 1949). Residual caprylic acid was analyzed using the HPLC method described by Herrera et al., 2009.

### 2.5 Microbiological quality control

Sterility was evaluated using the membrane filtration method (USP-NF, 2024c), where membranes were cultured in thioglycolate and soy trypticase media. Endotoxins were assessed using the Limulus amebocyte lysate (LAL) assay (USP-NF, 2024d; Solano et al., 2015). Pyrogens were evaluated using the rabbit model (USP-NF, 2024e; Solano et al., 2015).

### 2.6 Biological quality control

#### 2.6.1 General safety test

For the general safety test, two groups of ten individually identified CD-1 mice (female, 16–18 g) were used. The first group was injected intraperitoneally (IP) with 0.5 mL of antivenom. The second group (control group) was injected with 0.5 mL of sterile saline solution (SSE) instead of the antivenom. Each animal's weight was recorded daily for seven days after injection. The test was considered approved if individuals showed no unexpected reactions or signs of illness, survived, and maintained their weight by the end of the test period.

#### 2.6.2 Lethality neutralization

The efficacy of antivenoms in neutralizing snake venom lethality was assessed using a mouse model. Groups of five CD-1 mice (both sexes, 18–20 g) were pretreated with subcutaneous (SC) tramadol at a dose of 50 mg/kg to reduce pain during the test (Herrera et al., 2018). After 15 min, the mice received an intravenous injection of 0.2 mL of various dilutions of antivenom mixed with a constant challenge dose of venom (5 LD_50_s for *B. arietans* and *E. ocellatus* venoms, or 3 LD_50_s for *N. nigricollis* venom), using PBS as solvent. The mixtures were pre-incubated at 37 °C for 30 min. Groups of mice injected with mixtures where the antivenom was replaced by PBS were used as controls. The number of deaths in the following 6 h was recorded (Durán et al., 2021) to calculate the median effective dose (ED_50_) and its corresponding 95% confidence interval (95% CI) using Probits (Finney, 1971). The surviving mice were euthanized using CO_2_ inhalation.

### 2.7 Statistical analysis

For flux, pressure, yield, and turbidity values, the significance of differences between group mean values was assessed using the Kruskal Wallis test.

## 3 Results and discussion

### 3.1 Comparison of diatomites with different filterability profile

After being mixed with caprylic acid, the raw plasma is transformed into a suspension of bulky solids mainly composed of denatured albumin and caprylic acid, in an aqueous solution of immunoglobulins. At this point, the total protein in solution corresponds to the fraction of plasma protein that was recovered after caprylic acid precipitation, which is the material available to be recovered by DBF. The yield of DBF was calculated as the percentage of total protein in solution available before DBF that was recovered after DBF, using the equation specified in section 2.2. DBF does not induce important changes in the quali-quantitative composition of the proteins that remain in solution after caprylic acid precipitation.

Diatomites used in this study differ in their filterability properties. When arranged according to increasing permeability, decreasing surface area, and increasing pore size, diatomites followed this order: C65, C100, C300, and C1000. These diatomites were used to assist in separating the antivenom immunoglobulins from the precipitated plasma using a depth filter with a particle retention rate ranging between 10 and 30 μm.

**Fig. 1:**
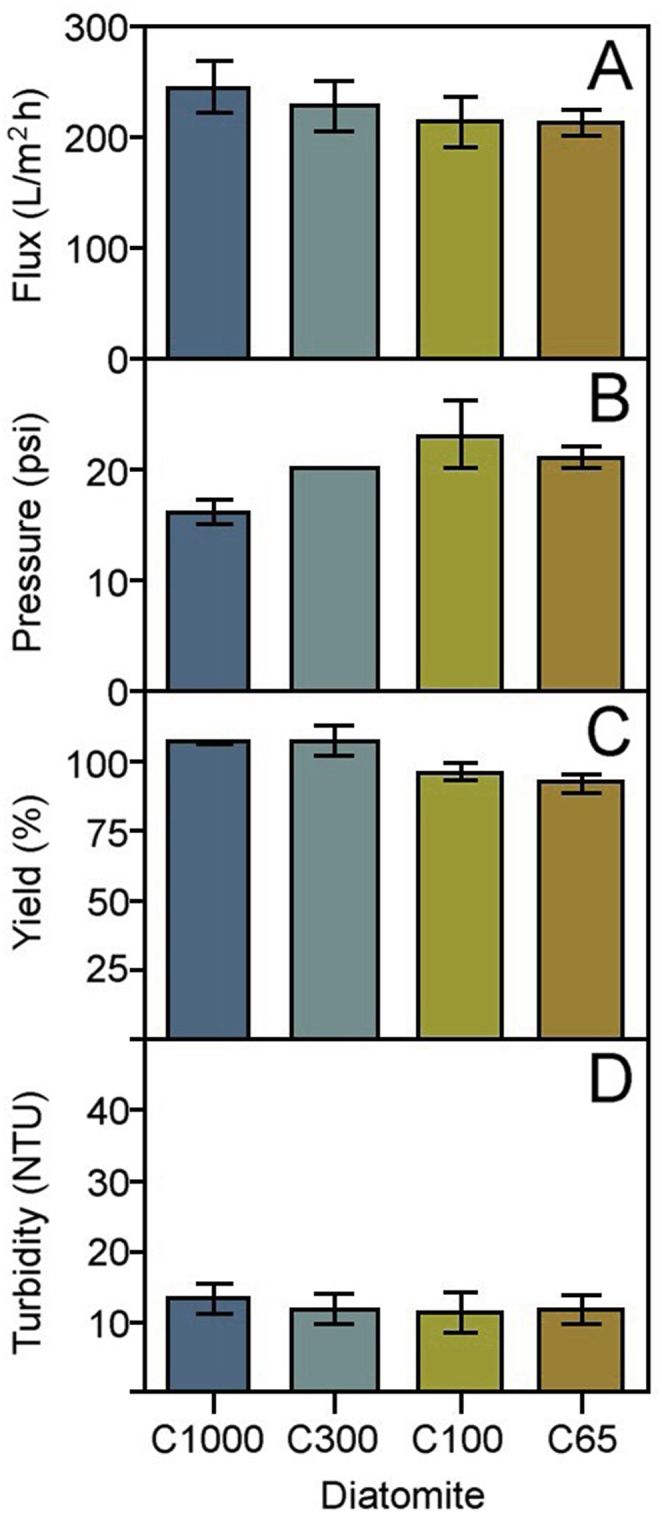
Comparison of different diatomites on the microfiltration of 100 mL of equine plasma precipitated with caprylic acid, showing: A) Maximum flux, B) Maximum system pressure, C) Yield, and D) Product turbidity. Results are presented as the average ± SD (n = 2). No significant differences were observed among the four types of diatomites for any variables.

Based on the presence of 30% solids in the precipitated plasma, the initial diatomite concentration was calculated as 90 g/L. In these experimental conditions, no significant differences were observed among diatomites regarding their effects on filtration flux (H _(3; 4)_ = 1.500, p = 0.682; Fig. 1A), system pressure (H _(3; 4)_ = 4.380, p = 0.224; Fig. 1B), process yield (H _(3; 4)_ = 5.500, p = 0.139; Fig. 1C), or the filtrate's turbidity (H _(3; 4)_ = 3.260, p = 0.353; Fig. 1D). C1000 diatomite was selected for pilot-scale experiments based on its overall performance (Fig. 1), despite the lack of significant statistical differences in the tested parameters.

**Fig. 2:**
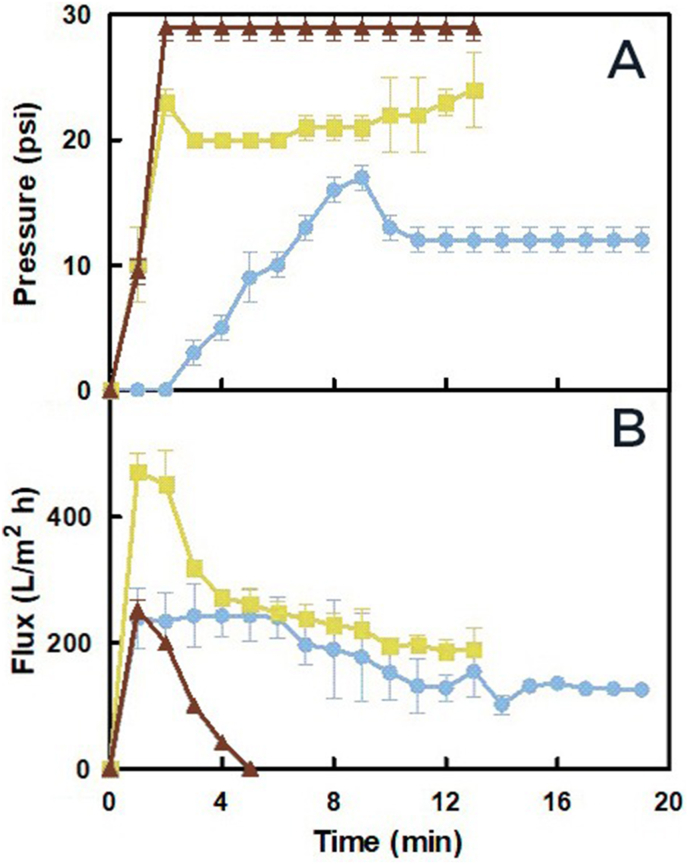
Comparison of different concentrations of C1000 diatomite on the microfiltration of 100 mL of equine plasma precipitated with caprylic acid, showing A) System pressure and B) Flux throughout the process. Brown triangles correspond to 30 g/L, green squares to 60 g/L, and sky-blue circles to 90 g/L. Results are presented as the average ± SD (n = 2).

To optimize the process, the performance of filtration aided by different amounts of C1000 diatomite (30, 60, and 90 g/L) was compared. When 30 g/L of diatomite was used, a rapid increase in pressure was observed, reaching the pressure limit of the filter capsule. In this instance, the flow ceased before completing the passage of the material (Fig. 2). When 60 g/L of diatomite was used, the capsule did not reach its pressure limit. The flux peaked around 1 min and then steadily decreased until the end of the process (Fig. 2). The filtration using 90 g/L of diatomite occurred at the lowest pressure among the tested conditions. In this case, the peak flux occurred at approximately 6 min (Fig. 2).

These results suggest that small particles remain unbound in the precipitated plasma at lower diatomite concentrations and accumulate within the flow channels of the depth filter. Consequently, a rapid increase in pressure was observed. Therefore, when small amounts of diatomite are used, more filtration surface area is required, increasing the cost of industrial-scale implementation. Based on these results, a concentration of 90 g/L in the precipitated plasma was selected for the pilot-scale experiments.

### 3.2 Performance of DBF at pilot scale

The combination of 50 L of equine plasma and caprylic acid resulted in the formation of a large precipitate. The solids accounted for 31 ± 1% of the mixture (Table 1). The expected filtrate volume (34.9 ± 0.9 L, Table 1) corresponds to the purified immunoglobulin solution, with a total protein concentration of 3.4 ± 0.3 g/dL. The total protein in the solution after caprylic acid precipitation was 1193 ± 62 g.

**Table 1:** DBF performance in the pilot-scale production of snake antivenoms.

Stage^a^	Analytical determination	Batch #1 (6600221)	Batch #2 (6610221)	Batch #3 (6620221)
1	Equine plasma (L)	51	50	50
	Plasma protein (g/dL)	7.2 ± 0.1	7.0 ± 0.2	7.5 ± 0.1
	Plasma ED_50_*E.ocellatus* (mg venom/mL antivenom)	1.1 (0.8–1.5)	1.4 (1.1–1.8)	1.4 (1.1–1.8)
	Caprylic acid (L)	3	3	3
2	Solids percentage (%)	30	30	32
	Solids (L)	15.3	15.0	16.0
	Expected filtrate volume (L)	35.7	35.0	34.0
	Filtrate protein concentration (g/dL)	3.2	3.4	3.7
	Total protein after caprylic acid (g)	1133	1190	1257
	Diatomite (kg)	4.6	4.6	4.5
3	Filtrate volume (L)	115	102	114
	Filtrate protein (g/dL)	1.1 ± 0.0	1.2 ± 0.0	1.2 ± 0.0
	Total protein after DE filtration (g)	1265	1224	1368
	Filtration yield (%)	112	103	109

a The unit operations that compose the caprylic acid method are: Raw material dispensing, caprylic acid precipitation, microfiltration, diafiltration, formulation, sterilization, aseptic filling in 10-mL vials, lyophilization, labeling and packaging. The samples in this table correspond to Stage 1: Before caprylic acid precipitation; Stage 2: After caprylic acid precipitation and before DBF; Stage 3: After DBF and before diafiltration.

The filtration of the precipitated plasma and diatomite mixture through the depth filter was completed in 30 min, instead of the more than 24 h required by an open filtration system (Rojas et al., 1994). The system pressure not exceeding 22 psi (1.5 bar). The filtrate containing immunoglobulins was transparent, with a faint bluish color due to traces of ceruloplasmin, a known contaminant in this procedure (Segura et al., 2012). After washing the protein filtrate trapped within the filter with 0.3 g/dL NaCl, the total volume of the recovered filtrate was 110 ± 7 L. The total protein content of this solution was 1286 ± 74 g, resulting in a yield of DBF of 108 ± 4%. The yield of the process and the organoleptic characteristics of the filtrates were no different from those obtained with an open filtration system (Rojas et al., 1994).

We obtained an average of 15 ± 2 vials of 10 mL per liter of plasma at the end of the purification process. The specific number in this context can vary based on the relationship between neutralizing potency of raw plasma and product specifications. Moreover, the process yield is affected by the loss of in-process product during each unit operation, including DBF, and the loss due to dead volumes in the equipment line.

### 3.3 Quality control of final products

The quality control of the three pilot batches shows that implementing DBF ensured the physicochemical characteristics of the formulation, compared to the product from our laboratory standard open filtration system. Residual moisture, reconstitution time, turbidity, and immunoglobulin monomer content meet product specifications. Similar results were found for pH and concentrations of NaCl, phenol, and sucrose. Osmolality and total protein levels, as well as residual caprylic acid, were within the expected range (Table 2).

**Table 2:** Quality control of three pilot-scale lyophilized antivenoms processed with C1000 diatomite during the filtration of the precipitated plasma.

	Batch #1 (6600221)	Batch #2 (6610221)	Batch #3 (6620221)
Residual humidity (%)	2.1 ± 0.6	1.6 ± 0.1	1.4 ± 0.2
Reconstitution time (s)^a^	79 ± 26	114 ± 106	107 ± 16
Turbidity (NTU)	33.0 ± 1.3	30.8 ± 1.2	32.5 ± 1.0
Monomers (%)	91.0	92.7	90.5
pH	7.03 ± 0.01	7.21 ± 0.01	6.90 ± 0.00
NaCl (g/dL)	0.84 ± 0.00	0.82 ± 0.00	0.82 ± 0.01
Phenol (g/dL)	0.16 ± 0.00	0.15 ± 0.00	0.15 ± 0.00
Sucrose (g/dL)	4.5 ± 0.1	4.8 ± 0.2	4.1 ± 0.1
Osmolality (mOsm/kg)	465 ± 2	455 ± 4	486 ± 2
Total protein (g/dL)	7.5 ± 0.0	7.4 ± 0.1	7.6 ± 0.1
Residual caprylic acid (ppm)	122 ± 3	91 ± 3	123 ± 3
Sterility (tioglycolate)	No growth	No growth	No growth
Sterility (soy trypticase)	No growth	No growth	No growth
Endotoxins (EU/mL)	<6.0	<3.0	<3.0
Pyrogens (Δ°C)	0.1 ± 0.1	0.1 ± 0.1	0.1 ± 0.1
General safety test	According	According	According
ED_50_*E. ocellatus* (mg/mL)	4.0 (3.2–5.7)	3.7 (2.8–5.4)	4.2 (3.5–5.6)
ED_50_*B. arietans* (mg/mL)	3.9 (3.0–5.1)	3.4 (2.7–4.4)	4.5 (3.5–6.5)
ED_50_*N. nigricollis* (mg/mL)	0.5 (0.3–0.7)	0.8 (0.7–1.3)	0.7 (0.5–1.0)

a Reconstitution time was reported as the average ± SD of six independent determinations.

The three batches passed the sterility test, demonstrating adequate management of areas, materials, and products during the process (Table 2). The endotoxin content in this formulation was below the bacterial endotoxin limit (11.6 EU/mL; Solano et al., 2015, Table 2). The low endotoxin concentrations in the three antivenom batches correlate with the minimal change in body temperature of rabbits during the pyrogen test (Table 2). Despite that similar results can be obtained with open filtration systems under controlled environmental conditions, the fact that DBF is performed in a single-use closed system in a short period of time ensures the microbiological quality of the primary clarification in a way that open systems cannot. This depurated process could help to prevent the contamination of antivenoms, which is an issue of concern (Solano et al., 2024).

All pilot batches passed the general safety test (Table 2), indicating that no toxic leachable impurities are released from diatomite or depth filters during DBF. The antivenoms also neutralized the venoms of *B. arietans*, *E. ocellatus*, and *N. nigricollis*, as indicated in the formulation design (Table 2).

Owing to its overall performance it was concluded that DBF could serve as a viable alternative for the GMP production of safe and effective snake antivenoms. The implementation of this technology on an industrial scale could enhance the productivity of antivenom producers. This could help alleviate the current shortage experienced by impoverished rural communities in low-income tropical countries (Basnyat and Shilpakar, 2022).

## Ethical statement

This manuscript presents an experimental study performed following the standard procedures of scientific ethics, including those related to the use and care of animals.

## CRediT authorship contribution statement

**Andrés Sánchez:** Writing – review & editing, Writing – original draft, Investigation, Conceptualization. **Maykel Cerdas:** Writing – review & editing, Investigation. **Jairo Gutiérrez:** Writing – review & editing, Investigation. **Mariángela Vargas:** Writing – review & editing, Investigation. **Álvaro Segura:** Writing – review & editing, Investigation. **María Herrera:** Writing – review & editing, Investigation. **Stephanie Chaves-Araya:** Writing – review & editing, Investigation. **Ronald Sánchez:** Writing – review & editing, Investigation. **Mauren Villalta:** Writing – review & editing, Investigation. **Gina Durán:** Writing – review & editing, Investigation. **Adriana Sánchez:** Writing – review & editing, Investigation. **Gabriela Solano:** Writing – review & editing, Investigation. **Daniel Cordero:** Writing – review & editing, Investigation. **Paola Sánchez:** Writing – review & editing, Investigation. **José María Gutiérrez:** Writing – review & editing, Writing – original draft, Project administration, Funding acquisition, Conceptualization. **Guillermo León:** Writing – review & editing, Writing – original draft, Project administration, Funding acquisition, Conceptualization.

## Declaration of competing interest

The authors declare the following financial interests/personal relationships which may be considered as potential competing interests: Guillermo Leon reports financial support was provided by 10.13039/100010269Wellcome Trust. If there are other authors, they declare that they have no known competing financial interests or personal relationships that could have appeared to influence the work reported in this paper.

## Data Availability

Data will be made available on request.
